# Supplementary material for: Zebrafish Melanoma-Derived Interstitial EVs Are Carriers of ncRNAs That Induce Inflammation
Source: Int J Mol Sci. 2022 May 14;23(10):5510. doi: 10.3390/ijms23105510 (PMC9143139; doi:10.3390/ijms23105510)
Supplement: Supplementary file 1 [file ijms-23-05510-s001.zip › ijms-1701822-supplementary/ijms-1701822-supplementary.pptx]

## Slide 1
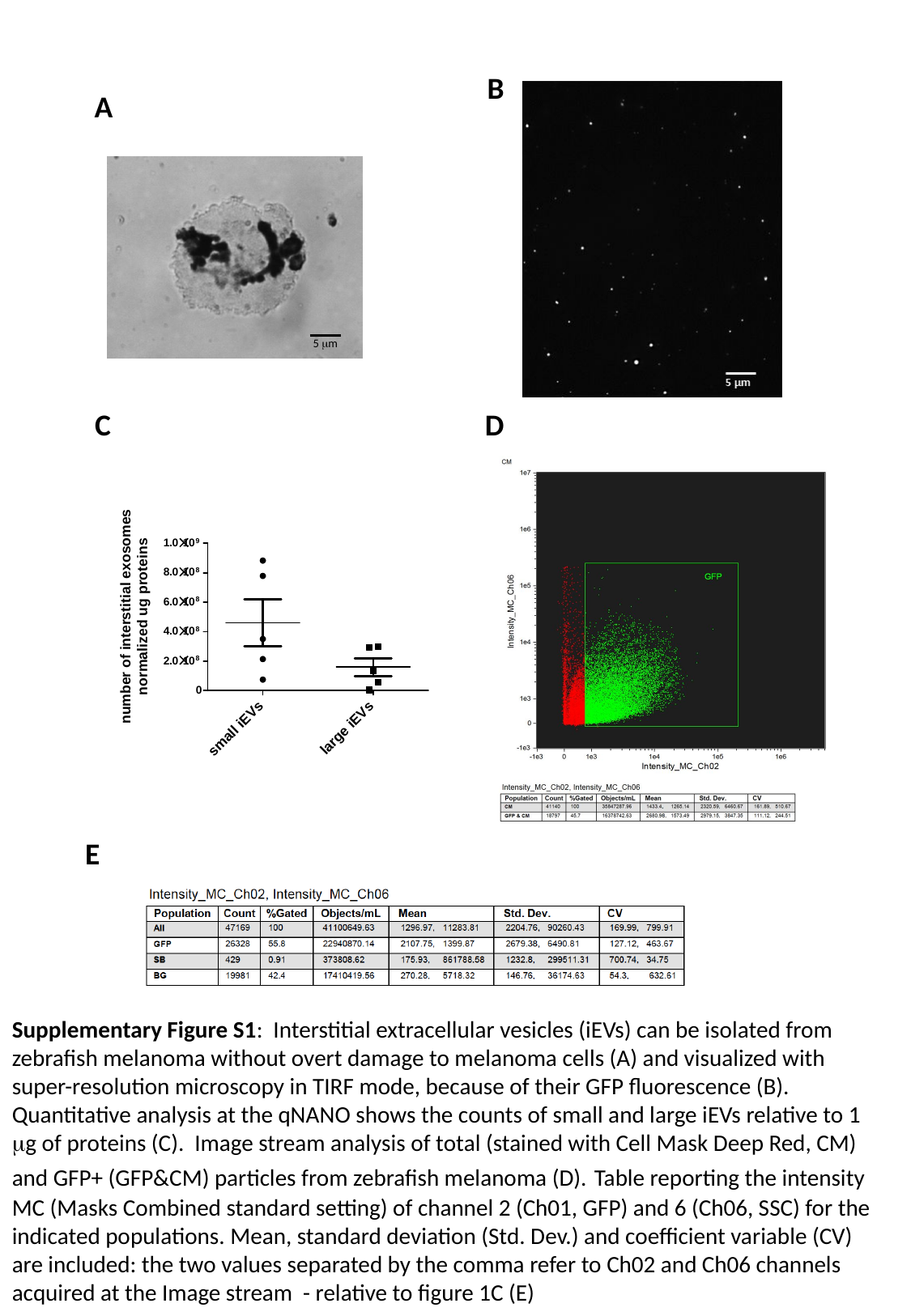

B
A
5 mm
C
D
E
Supplementary Figure S1: Interstitial extracellular vesicles (iEVs) can be isolated from zebrafish melanoma without overt damage to melanoma cells (A) and visualized with super-resolution microscopy in TIRF mode, because of their GFP fluorescence (B). Quantitative analysis at the qNANO shows the counts of small and large iEVs relative to 1 mg of proteins (C). Image stream analysis of total (stained with Cell Mask Deep Red, CM) and GFP+ (GFP&CM) particles from zebrafish melanoma (D). Table reporting the intensity MC (Masks Combined standard setting) of channel 2 (Ch01, GFP) and 6 (Ch06, SSC) for the indicated populations. Mean, standard deviation (Std. Dev.) and coefficient variable (CV) are included: the two values separated by the comma refer to Ch02 and Ch06 channels acquired at the Image stream - relative to figure 1C (E)

## Slide 2
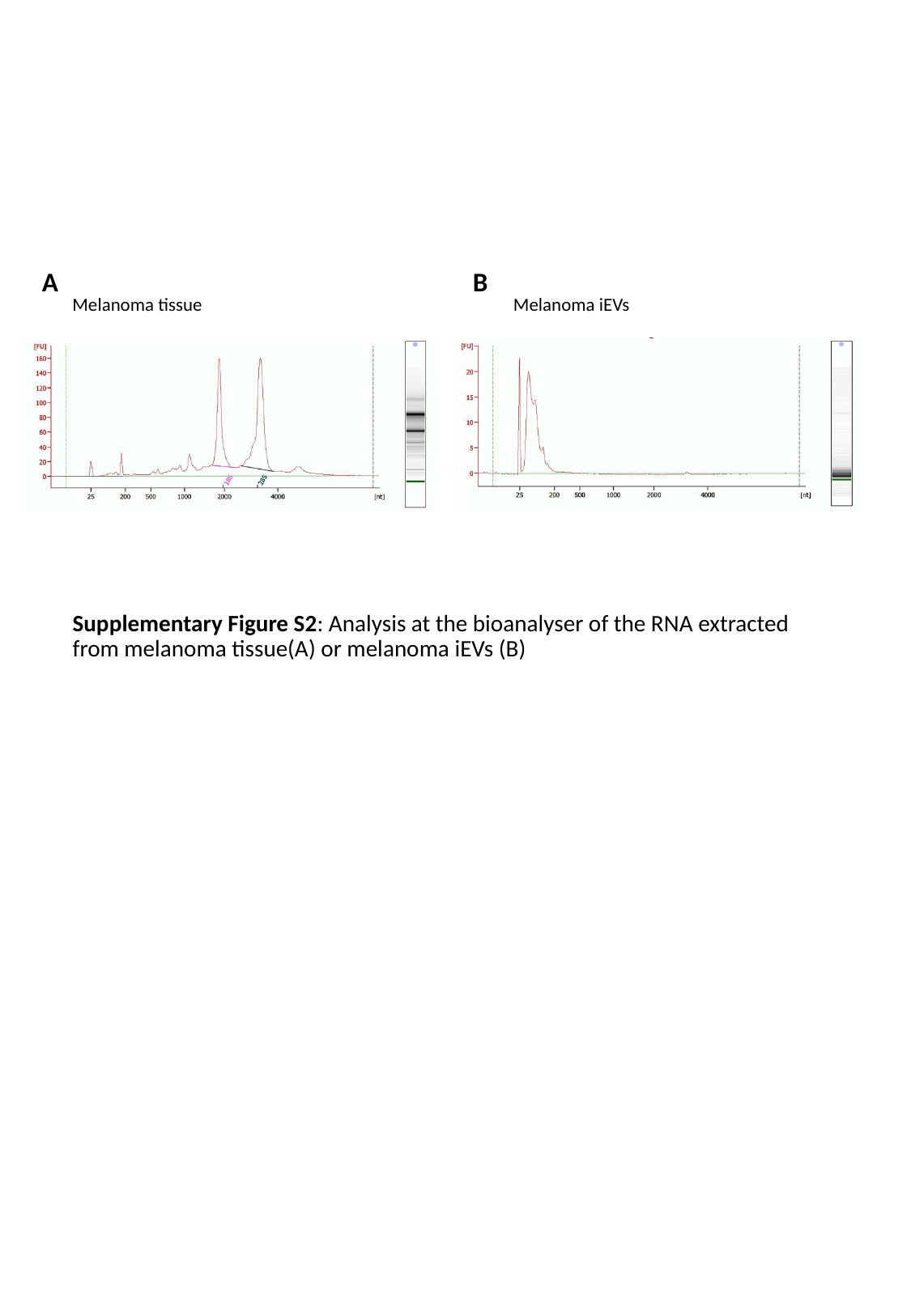

A
B
Melanoma tissue
Melanoma iEVs
# Supplementary Figure S2: Analysis at the bioanalyser of the RNA extracted from melanoma tissue(A) or melanoma iEVs (B)

## Slide 3
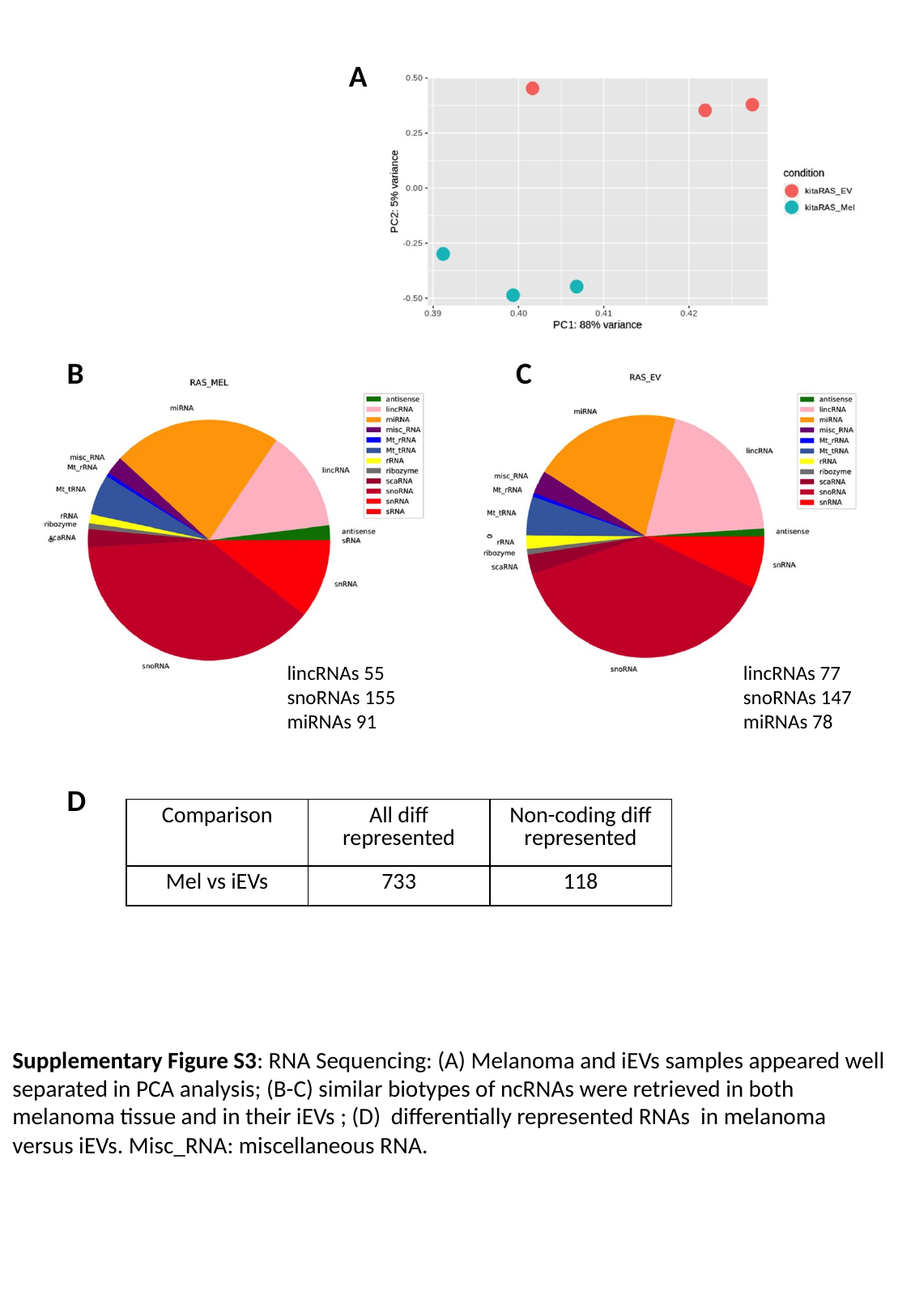

A
B
C
lincRNAs 77
snoRNAs 147
miRNAs 78
lincRNAs 55
snoRNAs 155
miRNAs 91
D
| Comparison | All diff represented | Non-coding diff represented |
| --- | --- | --- |
| Mel vs iEVs | 733 | 118 |
Supplementary Figure S3: RNA Sequencing: (A) Melanoma and iEVs samples appeared well separated in PCA analysis; (B-C) similar biotypes of ncRNAs were retrieved in both melanoma tissue and in their iEVs ; (D) differentially represented RNAs in melanoma versus iEVs. Misc_RNA: miscellaneous RNA.

## Slide 4
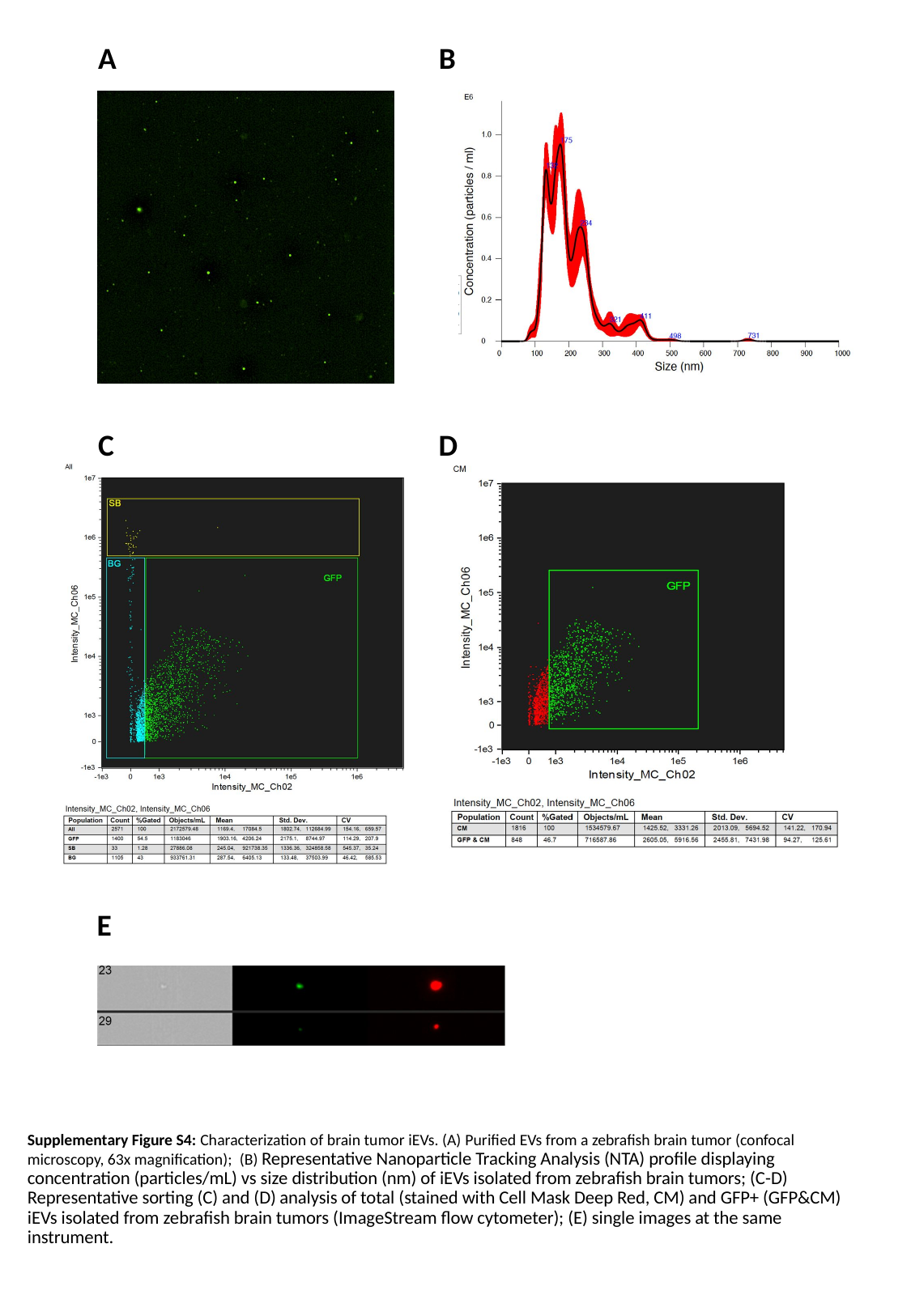

B
A
C
D
E
# Supplementary Figure S4: Characterization of brain tumor iEVs. (A) Purified EVs from a zebrafish brain tumor (confocal microscopy, 63x magnification); (B) Representative Nanoparticle Tracking Analysis (NTA) profile displaying concentration (particles/mL) vs size distribution (nm) of iEVs isolated from zebrafish brain tumors; (C-D) Representative sorting (C) and (D) analysis of total (stained with Cell Mask Deep Red, CM) and GFP+ (GFP&CM) iEVs isolated from zebrafish brain tumors (ImageStream flow cytometer); (E) single images at the same instrument.

## Slide 5
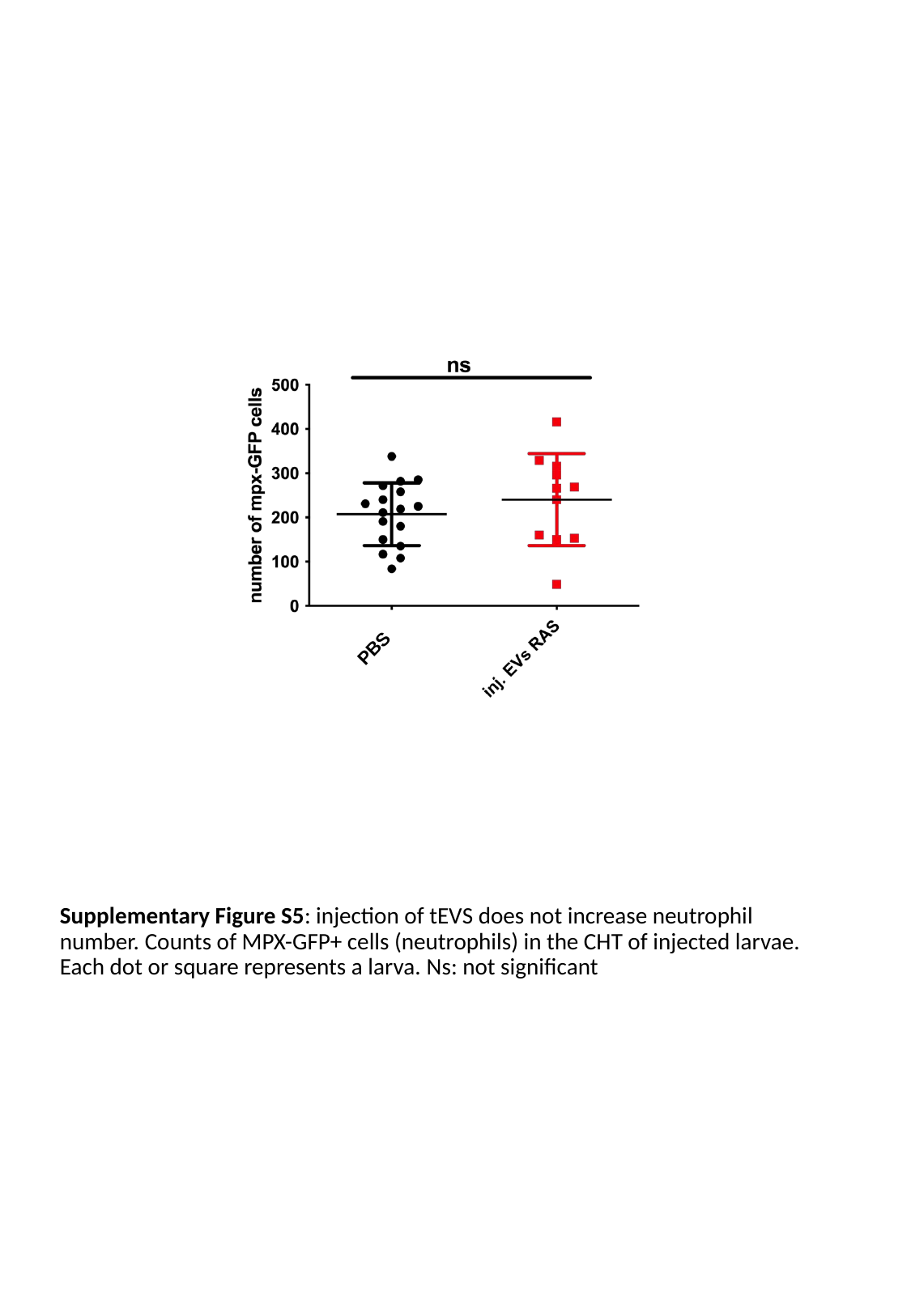

PBS
# Supplementary Figure S5: injection of tEVS does not increase neutrophil number. Counts of MPX-GFP+ cells (neutrophils) in the CHT of injected larvae. Each dot or square represents a larva. Ns: not significant

## Slide 6
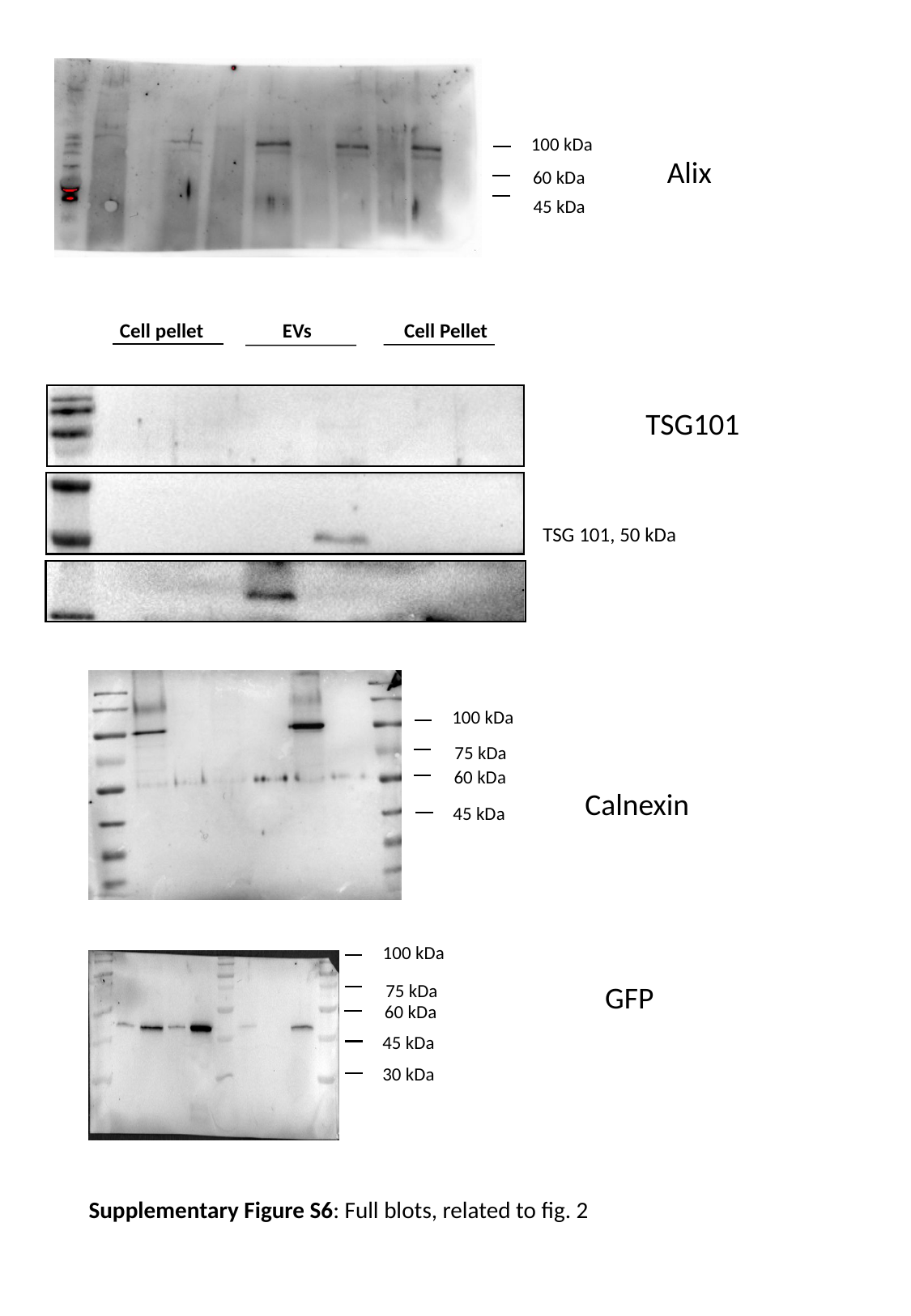

100 kDa
Alix
60 kDa
45 kDa
Cell pellet EVs Cell Pellet
TSG101
TSG 101, 50 kDa
100 kDa
75 kDa
60 kDa
Calnexin
45 kDa
100 kDa
75 kDa
GFP
60 kDa
45 kDa
30 kDa
Supplementary Figure S6: Full blots, related to fig. 2
